# Semi-Automated Approach for Retinal Tissue Differentiation

**DOI:** 10.1167/tvst.9.10.24

**Published:** 2020-09-23

**Authors:** Evgenii Kegeles, Tatiana Perepelkina, Petr Baranov

**Affiliations:** 1 The Schepens Eye Research Institute of Massachusetts Eye and Ear, Department of Ophthalmology, Harvard Medical School, Boston, MA, USA; 2 Moscow Institute of Physics and Technology, Dolgoprudniy, Russia

**Keywords:** retina, pluripotent cells, mouse embryonic stem cells, organoids, automation, forskolin

## Abstract

**Purpose:**

Three-dimensional strategy for the differentiation of pluripotent stem cells to the retina has been widely used to study retinal development, although the cell production and drug discovery applications are limited by the throughput. Here we attempted to scale up the protocol using a semiautomated approach.

**Methods:**

For the experiments we used the Rx-GFP mouse embryonic stem cell (mES) reporter cell line, specific for early retinal development and human embryonic stem cell line Brn3b-tdTomato, specific for retinal ganglion cells. To increase the throughput, we implemented automated media exchange using Thermo WellWash Versa with Thermo RapidStack robot. To analyze the rate of retinal differentiation in mouse stem-cell derived organoids we imaged the plates at day 10 of differentiation using Life Technologies EVOS Fl Auto. The automated image analysis of fluorescent images was performed with custom Python OpenCV script.

**Results:**

The implementation of a semiautomated approach significantly reduced the operator time needed: 34 minutes versus two hours for 960 organoids over the course of 25 days without any change in differentiation pattern and quantity of retinal differentiation. Automated image analysis showed that Forskolin treatment starting from day 1 leads to a significant increase in retinal field induction efficiency.

**Conclusions:**

Semiautomated approach can be applied to retinal tissue differentiation to increase the throughput of the protocol. We demonstrated that automated image analysis can be used to evaluate differentiation efficiency, as well as for troubleshooting and to study factors affecting retinal differentiation.

**Translational Relevance:**

Using robotic approach reduces the risk of human error and allows to perform all cycle of cell production in enclosed conditions, which is critical for GMP cell manufacture.

## Introduction

**Figure 1.**
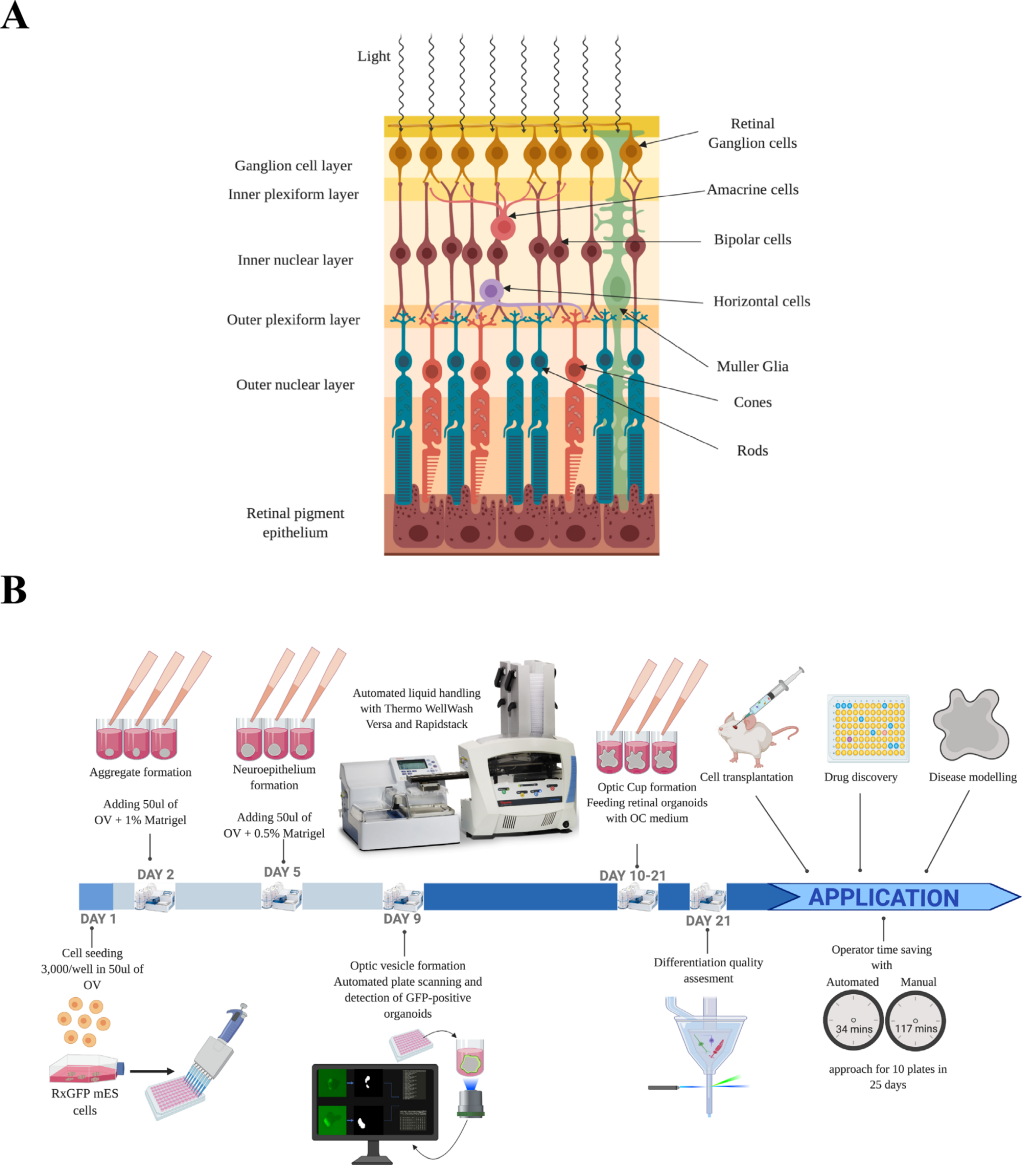
(A) Retinal structure. There are six main layers within the retina: RPE, which provides photoreceptors with nutrients and supports their function; photoreceptors (rods and cones, PR), which are located in the outer nuclear layer (ONL); HCs, which connect to the photoreceptors and form the outer plexiform layer (OPL) right adjacent to the ONL; bipolar cells (BC) and Muller glia (MG), which form the inner nuclear layer (INL)—the thickest layer of the retina; and amacrine cells and retinal ganglion cells, which form the inner plexiform layer (IPL) and ganglion cells layer (GCL), respectively. (B) Differentiation outline. Mouse embryonic cells are seeded in U-well plates with optic vesicle induction medium for the formation of aggregates, and the following day additional medium containing Matrigel as a matrix is added. Neuroepithelium forms on the periphery of the aggregate, and optic vesicles start stochastically appearing from it starting from around day 5, when an additional portion of Matrigel containing medium is added. Optic vesicles then continue to mature and form optic cups where the retina undergoes maturation and the retinal progenitor cells specify into retinal cell types. Automated liquid handling was implemented on days 2, 5, and 9 through 21. The organoids were imaged with an automated fluorescent microscope on day 9 for further image analysis. The organoids were collected on day 21 for the assessment of retinal differentiation with flow cytometry and confocal microscopy. This figure was created using Biorender.com.

The retina is a complex organ located in the posterior part of the eye and consists of the two main parts: the neural retina (NR) and the retinal pigment epithelium (RPE). The neural retina represents a set of highly organized layers of neurons connected together and forming a neuronal circuit that perceives light and processes incoming visual information before sending it to the brain. The structure, physiology, and development of mammalian retina is highly conserved and with six major cell types present: photoreceptors (rods and cones, PR), bipolar cells, and retinal ganglion cells (RGC) form vertical pathways, amacrine cells (AC) and horizontal cells (HC) allow for the modulation of a signal, and the Muller glia supports retinal architecture and maintains retinal homeostasis (Fig. 1A).

Because of the limited endogenous regenerative capacity in the mammalian retina and optic nerve, the loss of retinal neurons leads to progressive and irreversible blindness. Today more than 15 million people in the United States have glaucoma (almost 3 million^1^), age-related macular degeneration (about 10 million^2^), or other retinal degenerative disease. The epidemiologic situation is exacerbated by the overall aging of the population in the developed world, because aging is one of the most important risk factors.^3^^,^^4^ Different approaches for the treatment of retinal diseases are being developed: neuroprotection, gene therapy, cell replacement, and others. Although they differ in the mechanism of action, target disease, and methodology, all of them require a massive number of retinal cells for research and preclinical development.

First protocols for the differentiation of retinal neurons from pluripotent stem cells were based on two-dimensional differentiation of embryonic stem cells (ESCs) by directed guidance toward the desired cell types.^5^ The two-dimensional cultures allow focus on a single specific cell type, have an advantage of high purity, and are relatively easy to scale up; however, this approach does not match the in vivo development process and does not allow recapitulating of cell-cell interactions forming during normal development.^6^

The alternative strategy was proposed by Yoshiki Sasai's group, showing that retinal tissue can be obtained from mammalian pluripotent stem cells through the three-dimensional (3D) differentiation approach.^7^ This method recapitulates the in vivo development of the retina starting from aggregates of pluripotent stem cells, followed by the induction of neuroectoderm, spontaneous formation of optic vesicles,^8^ and followed by optic cup formation with retinal neuron specification and maturation. This approach results in the production of bona fide retinal neurons in a complex tissue in self-organized manner without ectopic stimulation of developmental pathways during the differentiation process. Resulting retina has striking metabolic, structural and physiological similarity to the normal retina,^9^ providing a robust and reproducible platform to model retinal diseases^10^ and to study retinal development.^11^

Despite all the advantages of the approach mentioned above, 3D differentiation method has its limitations: (1) the stochastic nature of the eye field induction—the efficiency varies from 30% to 68%^12^; (2) the time needed for proper recapitulation of retinal development—30 days for mouse retinal organoids^12^ and up to a year for human organoids^11^; and (3) heterogeneity—the retinal tissue contains multiple cell types, so an additional step is needed to isolate/enrich for photoreceptors or ganglion cells if they are the target. Here we attempted to address some of the problems mentioned above by implementing automation steps: automatic liquid handling and differentiation quality assessment using automated plate scanning with fluorescent microscopy and image analysis.

## Materials and Methods

### mES Cell Culture

The mouse embryonic stem cell (mES) reporter cell line RxGFP was used in this study (RIKEN).^13^ Cells were cultured in mES medium (Supplementary Table S1). Cells were fed every other day and passaged at 70% to 80% confluence on a cell culture–treated T-75 flask coated with 1% Matrigel (Corning Life Sciences, Corning, NY, USA) solution for one hour. For replating or seeding for retinal organoid formation, cells were dissociated using 0.25 Trypsin solution (Gibco, Waltham, Massachusetts, USA) for seven minutes at 37°C in a CO_2_ incubator.

### Differentiation of Mouse Retinal Organoids

**Figure 2.**
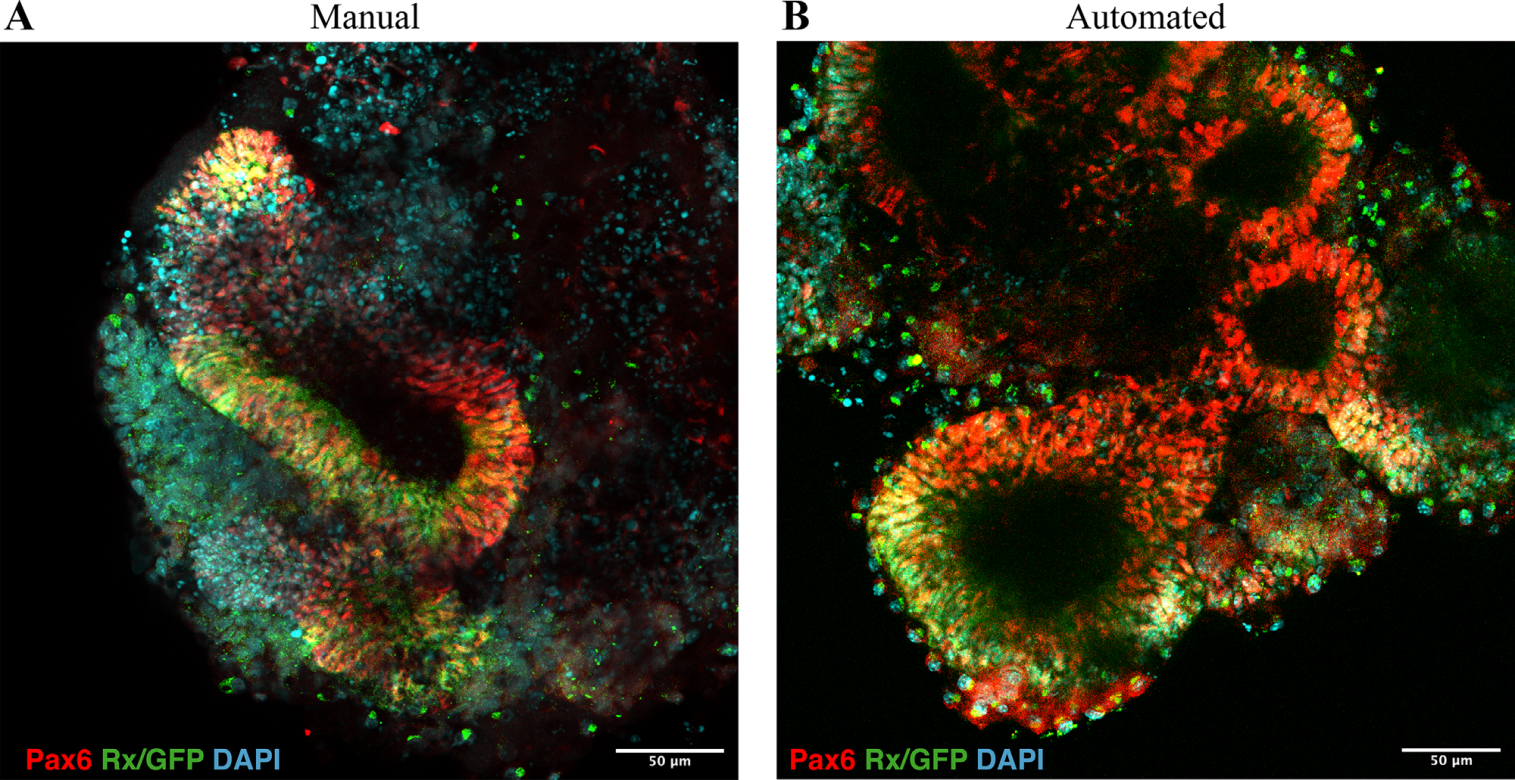
Automated liquid handling does not disrupt optic vesicle formation. Confocal images of retinal organoids at early development stage (day 10) with manual (A) and automated (B) liquid handling, stained for early retinal markers Rx, Pax6. *Scale bar*: 50 µm.

Differentiation was performed with the protocol reported before with minor modifications.^14^ The procedure is outlined in Figure 2. RxGFP mES cells were dissociated from the flask with 0.25 trypsin and seeded in differentiation medium (OV) (Supplementary Table S1) on 96-well U-bottom polystyrene plates (Greiner Bio-One, Kremsmünster, Austria) in cell density of 3000 cells per well in 50 µL of the media. Cell seeding was performed manually in both experimental groups. Cells were fed with 50 µL of OV supplemented with 1% Matrigel (Corning Life Sciences) manually or automated with WellWash Versa (Thermo Fisher, Waltham, MA, USA) depending on experimental group on day 2 of the differentiation, additional feeding with 50 µL of OV with 0.5% Matrigel was performed on day 5 of differentiation. Further medium change was performed in automated or manual manner with OC media (Supplementary Table S1) starting from day 9 every other day until the day 21.

### Differentiation of Human Retinal Organoids

To test whether the same automation strategy was able to maintain the differentiation of human pluripotent stem cells into retinal organoids, we used a Brn3b-tdTomato human embryonic stem cell reporter line (hES9).^15^ We used the same strategy as described for mouse cells with minor modifications: hES9 cells were cultured in mTESR1 media as colonies and passaged with Accutase every five days. For the differentiation we collected cells with collagenase 4 and resuspended them in the OV medium, supplemented with 5% KSR, 10uM Y-27632 and 1 µmol/L IWR-1-endo. Cells were plated at 5000 in 50 µL in 96-well U-bottom Corning ultralow attachment plates with either a manual multichannel pipette or through an automated liquid dispenser (WellWash Versa). After 24 hours, we added 50 µL of OV medium, and the medium exchange was performed the same way as described above for mouse cells. At days 9 and 12 we replaced 100 µL of medium with OC medium. At the day 14 we counted the number of organoids per plate with red fluorescence to check for retinal differentiation and collected organoids to visualize retinal ganglion cell differentiation.

### Functional Analysis of Mouse Stem-cell Derived Retinal Ganglion Cells

To test the organoids obtained through manual or automated liquid media exchange, we tested the ability of stem cell–derived retinal ganglion cells to respond to glutamate excitation. Retinal ganglion cells were isolated from mES-derived organoids at day 21 of differentiation by magnetic microbead-assisted sorting for Thy1 (CD90).^16^ The C90+ cells were replated on laminin-coated 16-well chamber glass slides in RGC medium (Neurobasal medium A, 1× B27, 5 µmol/L Forskolin, 50 ng/mL BDNF, 50 ng/mL CNTF, 1× Glutamax, 1× ITS-X) at 8000 cells per well and cultured for two days before functional assay. For the calcium analysis RGCs were loaded with Fluo-4 AM dye (Thermo Fisher): 1 mmol/L stock solution was diluted in BrainPhys medium to make 1 µmol/L loading solution. Cell medium was replaced completely with 100 µL of Fluo-4 AM working solution and cells were incubated at room temperature for 30 minutes in the dark. After 30 minutes slides with RGCs were washed with BrainPhys medium and filled with 100 µL of BrainPhys medium per well. Slides were imaged at inverted microscopy to detect Fluo-4 fluorescence before and after stimulation with glutamate (1 µmol/L final concentration). We counted the total number of cells per field of view and the number of cells responding to glutamate.

### Dissociation of Retinal Organoids

Papain (10 mg/mL) was added 1:100 to activation buffer (Supplementary Table S2) and incubated in an open tube in cell culture 7% CO_2_ incubator for 30 minutes to activate the enzyme. Retinal organoids were collected in a tube and washed with Hanks Balanced Salt Solution (HBSS) before the digestion. For dissociation the organoids were incubated at 37°C with activated papain solution for 15 minutes with agitation every two minutes. Finally, organoids were pipetted to resuspend cell clusters, washed with OC media, and strained with a 0.4-µm cell strainer.

### Flow Cytometry

One or two plates of retinal organoids were collected and dissociated with papain. Cells were fixed with 4% paraformaldehyde (PFA) for 30 minutes on ice. After the fixation, cells were washed with washing buffer and incubated in blocking buffer (Supplementary Table S2) for one hour on room temperature (RT). Staining with primary antibodies was performed in staining buffer (Supplementary Table S3) overnight at 4°C. The antibodies used are listed in Supplementary Table S3. The following day the cells were washed with washing buffer, and secondaries were applied in staining buffer for three to four hours on RT. For nuclear staining the samples were stained with NucBlue (Thermo Fisher) dye 15 minutes before the run on a MACS Quant (Mylteniy Biotec, Bergisch Gladbach, Germany) flow cytometer. Data analysis was performed with FlowJo software.

### Immunohistochemistry and Confocal Imaging

Ten to 15 organoids were collected and fixed with 4% PFA for 20 minutes on RT. Before staining they were blocked with Blocking buffer for one hour on RT. Staining with primary antibodies was performed overnight at 4°C in staining buffer. The next day, after being washed with wash buffer (Supplementary Table S2), the secondaries were applied overnight at 4°C. After antibodies staining and washing, the organoids were stained with DAPI for 10 minutes on RT and mounted on concavity slides (Lab Scientific, Livingston, NJ, USA). After that the samples were imaged with a Leica SP5 confocal microscope (Leica, Wetzlar, Germany). A few organoids from both groups were imaged, and then representative images from each of the group were selected.

### Plates Scanning and Image Processing

Organoids were scanned using the EVOS FL Auto (ThermoFisher) imaging system, which allows the scanning of 96-well plates in an automated manner. Resulting images were processed using a custom-developed Python script, which uses an OpenCV 4 library to work with images. For each image background was determined separately by averaging pixel intensities on a rectangle with the center in the center of the picture and dimensions equal 80% of the image's length and width. After the background subtraction, contrast adjustment was performed. Finally, image was thresholded, and total number of positive pixels was calculated as a picture score. The pseudocode is summarized below:

$$\begin{array}{ccc} background & = & average(image[rect(0.8\cdot \mathrm{height},\\ & & 0.8\cdot width)])\\ image & = & image-background\\ image & = & image\cdot \alpha +\beta \\ image\left[i\right] & = & 255\;if\;image\left[i\right]\geq threshold\;else\;0\\ score & = & count\left(image\left[i\right]=255\right) \end{array}$$

### Experimental Groups

In this study we compare manually and robotically handled retinal organoids. Organoids from both groups were seeded manually with a multichannel pipette. All of the following medium changes in the “automated” group were performed using WellWash Versa robot and in the “manual” group with a multichannel pipette.

### Statistical Analysis

For all comparisons between the experimental conditions, the nonparametric Mann-Whitney test was used.

## Results

### Optimization of Parameters for Automated Liquid Handling

To increase the throughput and consistency of the 3D differentiation protocol, we implemented robotic liquid handling on critical steps of the protocol: adding media on day 2 and day 5 and feeding on days 9 through 21 (Fig. 1B). To increase the consistency of differentiation and minimize the stress applied to the organoids due to the liquid change multiple parameters were adjusted: dispensing direction and height, aspiration height, aspiration offset and speed.

In order to match volumes which are being aspirated and dispensed by the machine to the existing differentiation protocol (Fig. 1B). We chose the slowest aspiration speed to increase the consistency of aspiration between wells and prevent aspiration of organoids from the wells. To find suitable aspiration height, we screened through possible positions and measured the volume left in the wells of a plate after aspiration gravimetrically (data not shown). We identified that the optimal aspiration height that leaves 100 µL of media in each well after aspiration is 7 mm, with the offset to the side of a well 1.3 mm (Supplementary Fig. S1B).

Furthermore, because organoids are fragile and susceptible to stress, it seemed to be important to dispense liquid on the wall of the well instead of dispensing directly toward the organoid in the center. This is why we tested two different wash heads available for the WellWash Versa: 1 × 8 wash head and 2 × 8 cell wash head. The default 1 × 8 wash head dispenser is directed vertically toward the center of the well while the cell wash head dispenses liquid to the side, which makes less impact on the organoid in the well (Supplementary Fig. S1A). Dispensing directly on the organoid caused a lot of damage, resulting in the mechanical disruption, evident by separate cell clusters in the well (data not shown). With the differentiation protocol we used, we expected organoid formation efficiency to be 100%,^14^ and with all the parameters optimized, automation did not affect this.

### Robotic Liquid Handling Does Not Disrupt Optic Vesicles Induction

Organoids in both experimental groups, handled manually and automatically, undergo normal retinal development as was assessed by IHC for early retinal markers. Figure 2 shows confocal images of optic vesicles formed in the organoids from both groups. These areas are characterized by the expression of eye field transcription factors which include Pax6 and Rx. Retinal Pax6+ areas have organized vesicle like structures with polarized planar tissue on the border. This tissue consists of retinal progenitor cells that are supposed to give rise to all the retinal cell types during the further development of the retina. Therefore automated liquid exchange does not disrupt optic vesicle induction in the organoids.

### Automated Liquid Handling Does Not Impede the Retinal Maturation in Mouse Organoids

**Figure 3.**
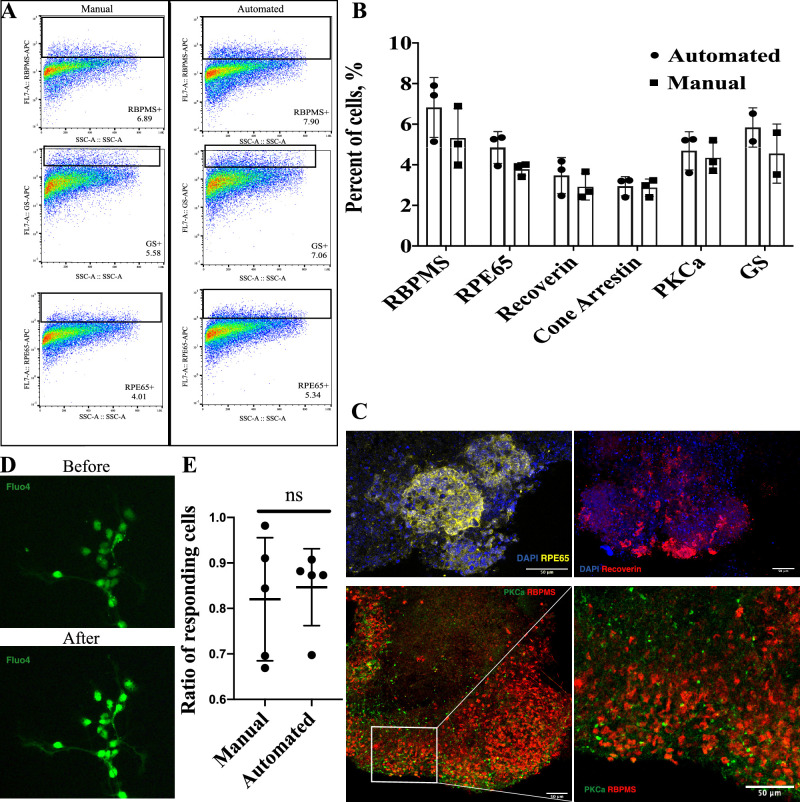
Automated liquid handling does not disrupt retinal tissue maturation. (A) Representative flow charts for cell-type specific markers show distinct populations of ganglion cells (RBPMS), Muller glia (GS), Retinal Pigment Epithelium (RPE65) in both groups. (B) The quantification of retinal differentiation outcome at day 21. Individual *dot* represents a single differentiation batch (biological replica), and data are shown as mean ± SD. Unpaired *t*-testing showed no difference in specific cell types as detected by flow cytometry: ganglion cells (5.3% ± 1.5% vs. 6.8% ± 1.5% RBPMS+, *P* = 0.275 in manual vs. automated group, respectively), retinal pigment epithelium (3.8% ± 0.3% vs. 4.8% ± 0.8% RPE65+, *P* = 0.095 in manual vs. automated group, respectively), photoreceptor progenitor cells (2.9% ± 0.7% vs. 3.5% ± 0.9% Recoverin+, *P* = 0.439 in manual vs. automated group, respectively), cone photoreceptors (2.9% ± 0.4% vs. 2.9% ± 0.5% Cone Arrestin+, *P* = 0.851 in manual vs. automated group, respectively), bipolar cells (4.4% ± 0.8% vs. 4.7% ± 0.9% PKCa+, *P* = 0.660 in manual vs. automated group, respectively), Muller glia (4.5% ± 1.5% vs. 5.8% ± 1.0% GS+, *P* = 0.408 in manual vs. automated group, respectively) (C) Immunohistochemistry of organoids in automated liquid handling group with confocal imaging shows normal pattern of differentiation and the formation of complex retinal tissue. Staining was performed for RPE65—retinal pigment epithelium marker (top left), Recoverin—photoreceptors (top right), PKCa—bipolar cells and RBPMS for retinal ganglion cells (bottom left and right). (D) Retinal ganglion cells isolated from mES-derived retinal organoids on day 21 before and after glutamate treatment. Calcium uptake was assessed using fluorescent calcium indicator—Fluo4 AM. (E) The fraction of RGCs responding to glutamate was calculated for each image from a fluorescent microscope. Each *dot* represents one field of view from the microscope.

Flow cytometry analysis was performed for the dissociated organoids on day 21 of the development to determine whether the automated liquid change had an effect on the yield of the different retinal cell types. Different cell types specific markers were used: RNA binding protein with multiple splicing (RBPMS) for retinal ganglion cells, PKCa for bipolar cells, Recoverin for photoreceptor precursors, Cone Arrestin for cones, GS for Muller glia, and RPE65 for RPE. The data are summarized in the Figures 3A and 3B. We found no significant difference in the yield of all the major retinal cell types mentioned above between the manual and automated experimental groups. We also observed presence of all expected retinal cell types with the predominance of RGCs (RBPMS) and small number of already appearing photoreceptors (Recoverin).

Also, IHC has be used to verify flow cytometry results and assess the tissue structure. As shown in Figure 3C (bottom left and right), the retinal organoids stained for RGCs marker RBPMS and bipolar cells marker PKCa contain retinal tissue located on the peripheral part of the organoids. Bipolar cells and ganglion cells are colocalized in the area, which shows that the retina is developing as a complex tissue. Staining for Recoverin showed that photoreceptors precursors started to appear as well (Fig. 3C, top right). Staining for RPE65 showed the presence of RPE patches on the surface of the organoids (Fig. 3C top left), which shows that not only neural retina was developing but RPE as well. Thus the robotic liquid handling does not disrupt the retinal tissue differentiation and maturation, which allows for derivation of mature retinal neurons.

### Robotic Liquid Exchange Does Not Affect the Functionality of Stem Cell Derived Retinal Ganglion Cells

To test the functionality of the cells derived from retinal organoids, we explored the ability of retinal ganglion cells to respond to glutamate. We isolated CD90-positive RGCs from organoids on day 21 from both experimental conditions—handled manually and with automation. As a measure of cellular response, we used calcium uptake, which could be visualized using fluorescent calcium indicator Fluo 4 AM. Cells before and after addition of glutamate are shown in Figure 3D. We did not find any significant difference in the ratio of cells responding to glutamate between our experimental groups: 82% ± 14% versus 85% ± 8% of cells responding per field of view in manual versus automated experimental groups (Fig. 3E). Thus the automated differentiation procedure allows us to derive functional retinal neurons from pluripotent stem cells.

### Operator Time Saving With the Automated Liquid Handling

To compare an operator time needed for feeding a batch of 10 plates of organoids manually and with the machine, we measured the time needed for a person to feed 10 plates and compared this time with the time needed to set up the machine. For adding the media on days 1, 2, and 5, it takes approximately six minutes for 10 plates; for feeding organoids it takes approximately 11 minutes for 10 plates. To connect the tubing and prime a machine, it takes approximately 2.5 minutes of operator time. The robot work time is 3.5 minutes for adding and 6.5 minutes for feeding. As a result, for 10 plates in a course of 25 days, it requires 34 minutes of operator time for automated culture and 117 minutes for manual cell culture. Furthermore, operator time does not change in case of the scaling up the protocol, whereas with manual culture, the time requirement increases proportionally.

### Automated Plate Scanning and Reporter Fluorescence Quantification Could Be Used to Assess the Retinal Differentiation Quality

The retinal differentiation in 3D is spontaneous—it is not possible to predict in which part of the organoid it will take place: the eye field randomly protrudes from the neuroectoderm, with the number and the size of the retinal areas varying from organoid to organoid within the batch. This variability warrants the inclusion of a quality control step into the differentiation pipeline. The manual selection of “good” organoids based on the reporter fluorescence is limited in throughput and does not allow us to objectively assess the differentiation batch.

**Figure 4.**
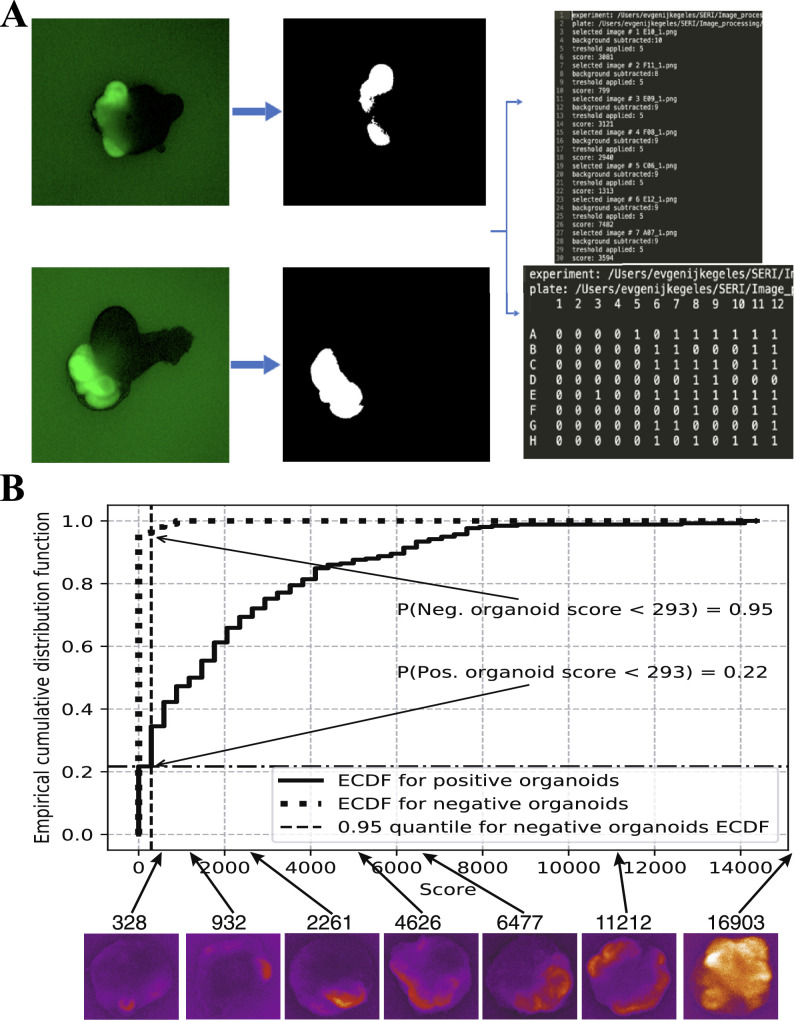
Automated plates scanning, and fluorescence analysis can be used to detect retinal areas in stem cell derived organoids. (A) Procedure overview: the program receives fluorescence images on input, extracts the positive area, and returns the score calculated for each organoid in a batch and layout of positive organoids within each plate. (B) Empirical cumulative probability functions for datasets of manually selected positive and negative organoids and 95% quantile for negative organoids distribution as a threshold. Representative organoids corresponding to each score are shown under the graph.

To address the problems mentioned above, we implemented automated plate scanning and developed a script (available at: https://github.com/zhenyakeg/GFP_fluorescence_detector) for the detection of fluorescent reporter driven under the promoter of the eye field–specific gene Rx. Using the program, we could extract the size of GFP-positive areas for every organoid in a batch and return the value (Score), which corresponded to the size of the developing retina. The user can determine a threshold that can be used to select “positive” organoids and program returns the layout of positive organoids for each plate which has been processed. The pipeline is summarized in Figure 4A.

**Figure 5.**
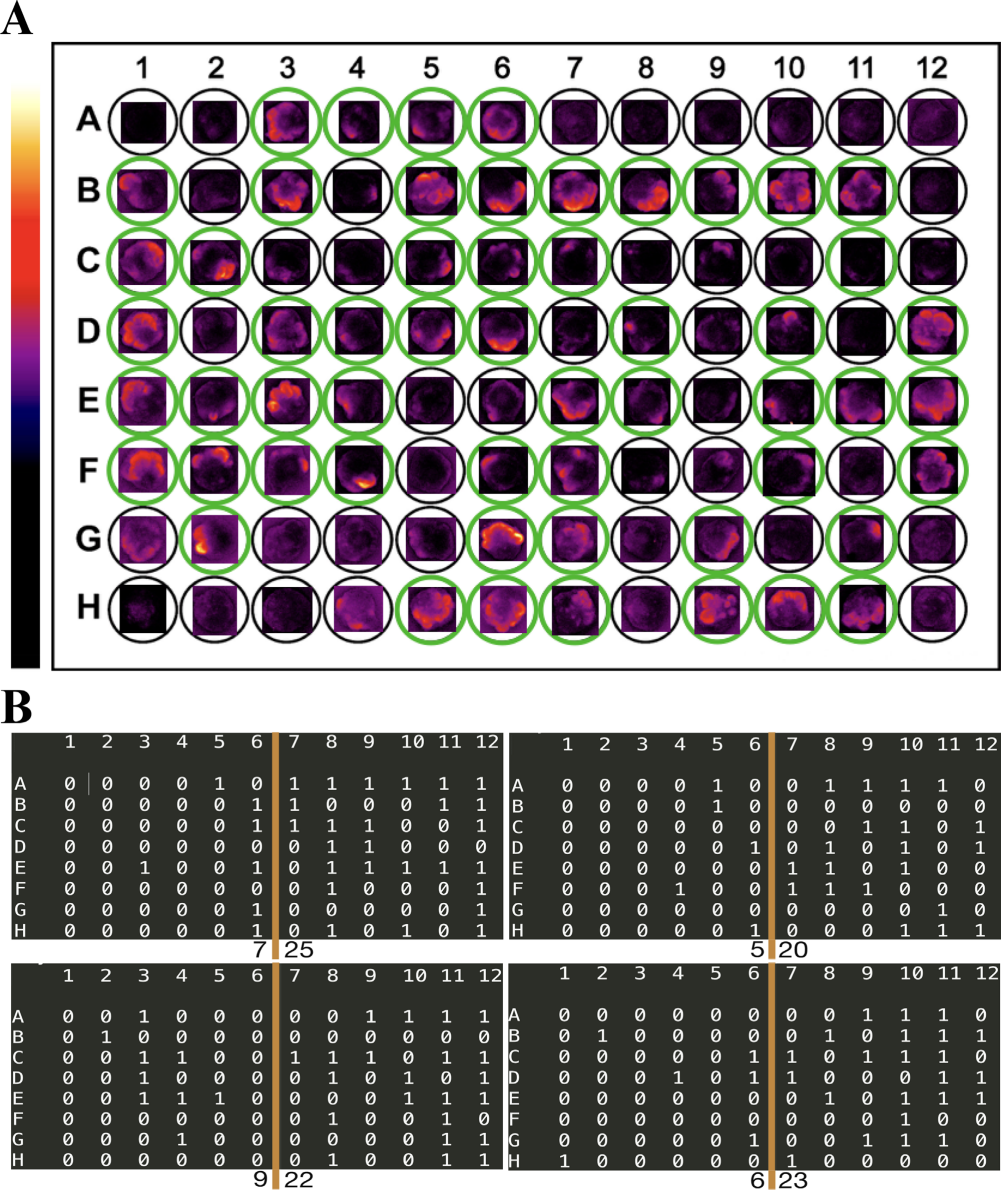
Fluorescence detection could be useful for troubleshooting or quality control of retinal differentiation. (A) Example of a plate scanned and analyzed on day 10 with the script; *green circles* show selected organoids, lookup table (LUT) is shown on the left side of the plate. (B) The plates were scanned and analyzed to detect fluorescence areas. The number of positive organoids on both sides of each plate has been calculated and represented as a number under each half of the plate layout in the figure. The layout shows that there are more retina positive organoids on one side of a plate than on the other.

Using the RxGFP reporter cell line and this computational tool, we attempted to identify positive and negative organoids within the differentiation batch. To do so, we manually classified ∼500 organoids into retinal and nonretinal categories on the basis of GFP fluorescence on day 10 of development. The resulting datasets were analyzed with the script, and the score for each organoid was calculated. To determine the threshold, we calculated a cumulative empirical distribution function for each dataset separately (Fig. 4B), and the threshold was determined as the score corresponding to the 95% quantile of negative organoids distribution. For the RxGFP cell line on day 10, we determined that the threshold score was 293, which corresponds to ∼1.3% of the total organoid projected area. Therefore organoids with a score exceeding the threshold represent a population that does not contain more than 5% of negative organoids. An example of the scanned plate and organoids that the program selected is shown in Figure 5A.

Furthermore, fluorescence analysis could be helpful in the search of differentiation enhancers, as well as troubleshooting. In one of the differentiation batches, we noticed that the differentiation rate on one side of a plate was significantly lower than on another (6.8 ± 1.7 vs. 22.5 ± 2.1, *P* < 0.0001 organoids per half of a plate) (Fig. 5B). This most likely happened because the organoids located closer to the incubator door were exposed to lower temperature than others, which caused them to lag in development. Such a pattern is almost impossible to notice looking at one organoid at a time through a microscope.

**Figure 6.**
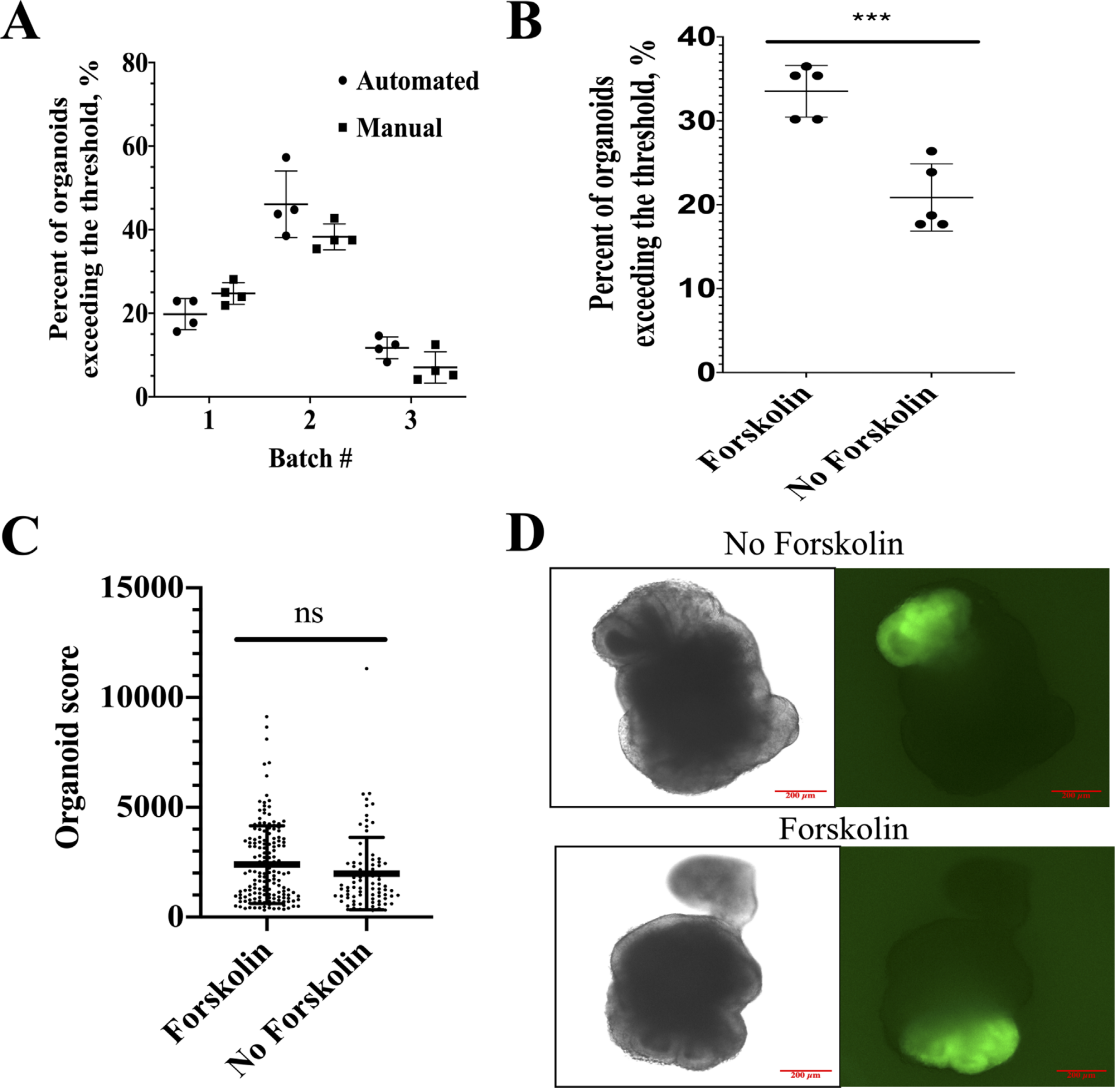
Automated organoids selection can be used as a readout to evaluate an effect of different conditions on retinal differentiation. (A) The proportion of positive organoids has been calculated for automated and manual groups for four plates in three different differentiation batches (mean and SD showed in the figure). Each *dot* on the graph represents a single plate of organoids. Unpaired *t*-testing showed no difference between automated and manual experimental groups within each batch: for the batch no. 1 (24.7% ± 2.6% vs. 19.8% ± 3.7% retina positive organoids, *P* = 0.072 in manual vs. automated group, respectively), batch no. 2 (38.3% ± 3.1% vs. 46.1% ± 8.0% retina positive organoids, *P* = 0.117 in manual vs. automated group, respectively), and batch no. 3 (7.0% ± 3.7% vs. 11.7% ± 2.6% retina positive organoids, *P* = 0.086 in manual vs. automated group, respectively). (B) To assess an effect of addition of Forskolin on early retinal differentiation five plates (biological replicas) were scanned on day 7 and analyzed using the script for fluorescence detection. Each *dot* on the graph represents a single plate. Unpaired *t*-testing showed significant increase in the rate of retinal differentiation in the group treated with Forskolin: 33.5% ± 3.1% vs. 20.9% ± 4.0% retina positive organoids, *P* = 0.0006 in Forskolin treated versus not treated, respectively. (C) Rx+ areas were calculated for both treated and nontreated experimental groups (mean and SD showed in the figure). Unpaired *t*-test showed no significant difference between treated and nontreated experimental groups (2390 ± 1770 vs. 1985 ± 1651 score, *P* = 0.0847 in treated vs. nontreated groups, respectively). Each *dot* on the graph represents the single organoid. (D) Representative images of retina-positive organoids from the Forskolin-treated and -nontreated experimental groups. Positive organoids from both groups show a similar differentiation pattern.

Ratios of positive organoids are variable among different differentiation batches as shown in Figure 6A. Automation has not improved the consistency within the batch, which could mean that the source of the difference between organoids is in spontaneous differentiation and not in handling.

### Automated Fluorescence Analysis Revealed an Effect of Forskolin on Early Retinal Development

We used our fluorescence analysis approach to evaluate an effect of Forskolin on early retinal differentiation efficiency. Forskolin was added to differentiation media starting from day 1 in concentrations 10 µmol/L. Treated and nontreated organoids were compared on the rate of retinal differentiation on day 7 of differentiation. We found that Forskolin treatment increased the rate of retinal differentiation: 33.5% ± 3.1% versus 20.9% ± 4.0% retina positive organoids on day 7 (*P* = 0.0006) for treated and nontreated groups, respectively (Fig. 6B). Interestingly, it did not change the differentiation pattern: the retinal area in positive organoids did not change significantly as calculated with the script (Fig. 6C), and the structure of optic vesicles in positive organoids was also preserved (Fig. 6D).

### Automated Differentiation Strategy Was Able to Maintain the Differentiation of Human Pluripotent Stem Cells into Retinal Organoids

**Figure 7.**
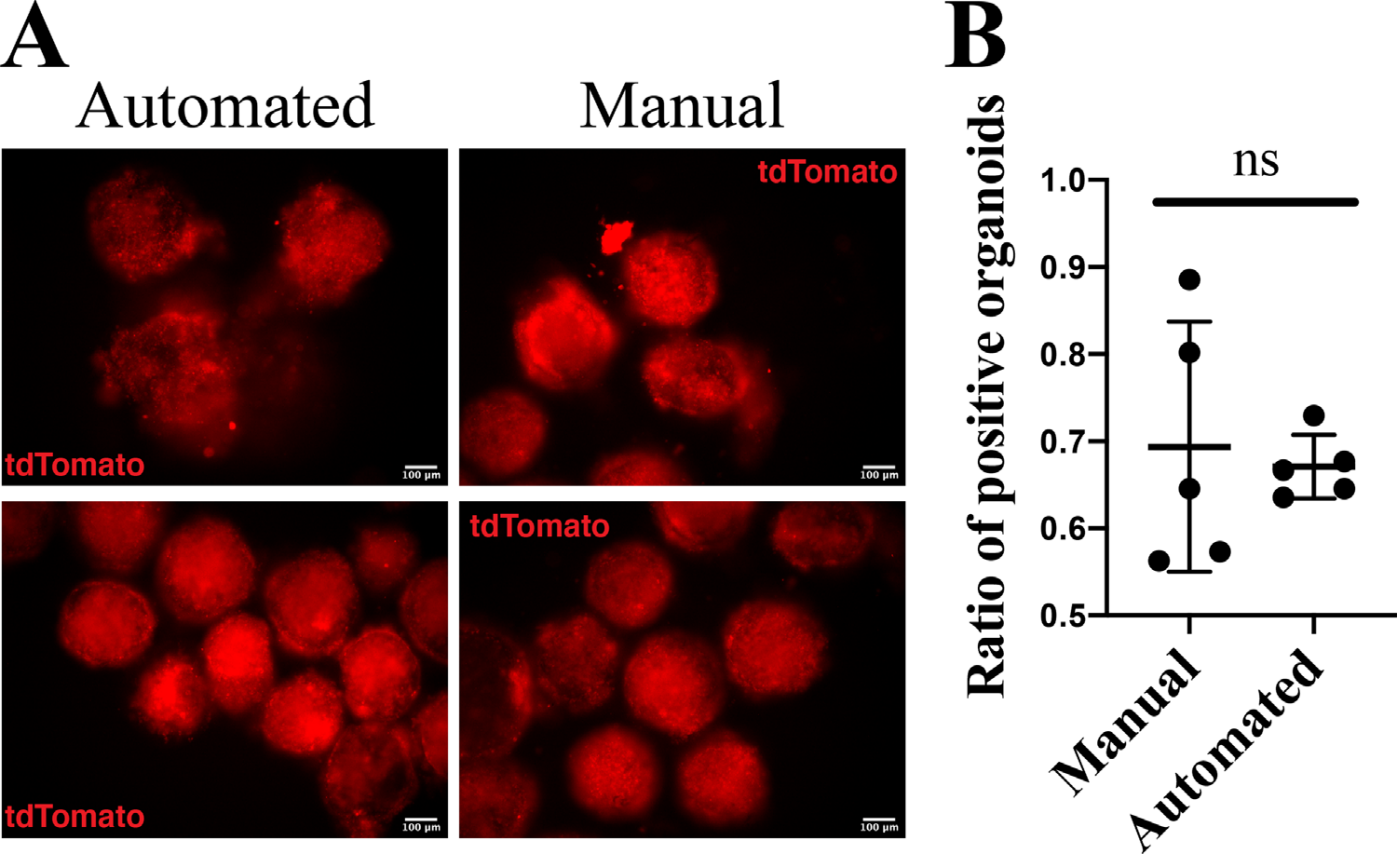
Automated differentiation strategy is able to maintain the differentiation of human pluripotent stem cells into retinal organoids. (A) Representative images of human Brn3b-tdTomato organoids on day 14 handled with the automated and manual protocols. Organoids from both groups have multiple tdTomato+ areas, which shows that automation does not affect retinal differentiation and emergence of retinal ganglion cells. (B) To compare the differentiation efficiency of manual and robotic approach for human stem cells, the number of tdTomato-positive organoids was calculated for each differentiation condition. Each *dot* on the graph represents a single 96-well plate of developing organoids. Nonparametric Mann-Whitney statistical testing did not show any significant difference between two experimental groups: 69% ± 14% versus 67% ± 4% tdTomato-positive organoids for manual versus automated conditions.

To test whether the same differentiation strategy was translatable to differentiation of human pluripotent stem cells, we used a Brn3b-tdTomato human embryonic stem cell reporter line. Organoids in both experimental groups, handled manually or with automation, were differentiated until day 14 of development, when the retinal ganglion cells already appearing could be assessed by the expression of a Brn3b-tdTomato fluorescent reporter (Fig. 7A). Organoids from both groups had multiple tdTomato+ areas, which shows that automation does not affect retinal differentiation and emergence of retinal ganglion cells. There was no statistically significant difference found in the proportion of tdTomato-positive organoids between the automated and manual conditions: 69% ± 14% versus 67% ± 4% in manual versus automated groups, respectively (Fig. 7B). This indicated that the robotic liquid exchange did not impede retinal differentiation in human organoids.

## Discussion

Organoid cell culture has become an important technique for the differentiation of stem cells into the tissues of different lineages: brain and retina, lung, intestine, kidney, etc.^17^ The success of this approach in getting complex heterogenous in vivo–like systems with proper tissue architecture is based on the recapitulation of normal development. In case of retinal differentiation, cells undergo the whole differentiation process starting from the aggregate of stem cells to the fully mature retina. During the process, cells form the neuroepithelium where the spontaneous eye field induction happens. Retinal progenitor cells from the eye field further differentiate forming all retinal cell types. This approach does not rely on any extrinsic stimulation of developmental pathways or genetic modification of embryonic stem cells, which implies that cell fate determination is orchestrated by the intrinsic signals and cell-cell interactions within the organoid.

One of the challenges that we aimed to address in our work is the ability to scale up the differentiation to produce sufficient numbers of organoids for drug discovery and cells for cell transplantation experiments. The current state of the art for scale up involves the use of rotary and spinning flask bioreactors. For human organoids, the use of bioreactors helped to increase the yield of photoreceptors 1.5 times compared to the conventional suspension Petri dish and showed improved proliferation of retinal progenitors and a decrease in apoptosis.^18^ This approach addresses the problem of low yield from the perspective of increasing viability of the maturating neurons in the organoids by improving the supply of nutrients and oxygen to the organoids.^19^ Pooling organoids in larger vessel minimizes human error, evaporation, and stress to the organoids, because of the larger medium volume and lower amount of handling in comparison to multiwell plates, but it makes it impossible to use each separate retinal organoid as a biological replicate. We used automated liquid handling in 96-well plates during the course of differentiation of mouse embryonic stem cells into retina (21 days).

Moreover, in addition to previously reported variability in differentiation efficiency among different mouse pluripotent stem cell lines (eye field induction efficiency varies between 31%-68%)^12^ and for human cells,^20^ we observed high differences in differentiation rate between different seeding batches within one cell line (7%-46%, relative standard deviation [SD] = 56%). However, differentiation rate was similar between the plates within one batch in either the manually or automatedly handled groups (relative SD = 22%). This allows us to assume that the differentiation is highly dependent on the state of initial pluripotent stem cells before the beginning of the differentiation process. This point of view can be additionally supported by indications of heterogeneity in stem cell populations, which was previously reported.^21^^,^^22^ So, probably implementation of additional assessment steps before the beginning of differentiation, which can predict the best state of mES cells for the beginning of differentiation, may be helpful to eliminate the observed batch effect, especially taking into account the fact that recent advancements in the deep learning field have made it possible to predict an onset of spontaneous differentiation of mES culture with high precision.^23^

In this study we developed an approach for scoring the level of retinal differentiation in the organoids using RxGFP reporter cell line with automated fluorescence plates imaging and image analysis. This approach allows us to implement fast, automated, and “noninvasive” readout during the early step of differentiation, which makes it possible to grade organoids based on the size of the retinal area on the early stage of development. Having an easy readout to look into differentiation efficiency allows for the optimization of the protocol or troubleshooting.

For instance, we could check an effect of Forskolin—an activator of adenylate cyclase, which was previously reported to increase the survival and neurite outgrowth in retinal ganglion cells^24^—and add it to the differentiation media on early step of differentiation, which was previously shown to increase the expression of eye field transcription factors and improve the yield of RGCs in retinal organodis.^15^ Here we were able to look at the eye field area in each organoid separately and prove that Forskolin, indeed, increases the rate of eye field induction. However, interestingly it has no effect on the size of the retinal area or structure in each separate organoid, showing that although it has an effect on differentiation efficiency, it does not have an effect on differentiation pattern. Previously high throughput imaging and automated analysis were also successfully used for optimization of differentiation conditions in kidney organoids^25^; researchers could identify optimal concentration of kinase inhibitor CHIR99021 for differentiation induction and seeding cell density for multiple cell lines. Their findings show that optimal parameters for the protocol vary between tested cell lines. Probably, differentiation efficiency between different cell lines that has been shown before for retinal organoids^12^ can be also minimized by the adjustment of differentiation conditions.

Moreover, this approach of the fluorescence quantification is not restricted in application to the reporter cell lines, but can be also used for the analysis of the physiological state of the cells within the organoid.^26^ For example, it has been shown before that fluorescence quantification can detect oxidative stress caused by hydrogen peroxide treatment with the staining dihydroethidium (DHE) or mitochondrial depolarization with JC-1 dye.^26^ This shows a high range of possible applications of the high-throughput fluorescence quantification approach in multiple assays.

Organoid cell culture is an appealing system for drug screening because it has a much higher throughput in comparison to conventional animal models, and it has a much closer cell-cell interaction and tissue architecture to the real organ.^27^ However, for high-throughput screening compatibility, a very important parameter to be considered is the low variability between samples on which testing would be performed because internal variability will complicate the interpretation of the results and distinguishing a real effect from the drug and stochastic variability between treatment subjects. This is why drug screening works so well for cancer 3D organoids, where organoids are formed very efficiently with high reproducibility.^28^ Using retinal organoids for high throughput drug discovery has also been proposed.^29^ However, we have shown for retinal organoids that the size and number of eye fields/optic vesicles varied a lot from organoid to organoid in a batch (scores were in a range between 300 and 17,000 because of the stochastic nature of eye field induction, hampering application of the technology for high throughput drug screening. Nevertheless, we suppose that our grading approach based on the retinal area could help to overcome this issue. Having the information about the score of each single organoid in a batch, it would be possible to compare effects of different therapeutics on organoids that sharing close scores. This still will not help to decrease the need in high amounts of organoids to perform the screening, but in combination with high throughput organoids production, it could become a promising approach for a drug discovery for retinal diseases.

Heterogeneity among organoids and batches also bring limitations for the use of 3D organoids for disease modeling,^30^ because it complicates the separation of effects caused by the differentiation process and, for example, different genetic backgrounds in case of using patient-specific iPSC cell lines. Our grading system allows us to determine the best performing organoids that have the largest retinal areas and can be studied, for example, for cell-cell interaction and function, and this data can be then compared between experimental groups.

Finally, we postulate that retinal area can be the most important and relevant parameter to estimation the quality of retinal differentiation in the organoid. Additional parameters may include more complex readouts (e.g., the presence of RPE, lamination of the neuroepithelium, etc.).

## Conclusion

Here we implemented a robotic liquid handling into 3D retinal differentiation protocol and showed that it had no negative effect on the differentiation outcome. Also, we developed a tool for automated detection of retinal areas and grading organoids on the size of the eye field and showed its application for differentiation conditions optimization, quality control during the differentiation, and troubleshooting.

## Supplementary Material

Supplement 1

Supplement 2

Supplement 3

Supplement 4
