# Supplementary material for: Semi-Automated Approach for Retinal Tissue Differentiation
Source: Transl Vis Sci Technol. 2020 Sep 23;9(10):24. doi: 10.1167/tvst.9.10.24 (PMC7521179; doi:10.1167/tvst.9.10.24)
Supplement: Supplement 1 [file tvst-9-10-24_s001.pdf]

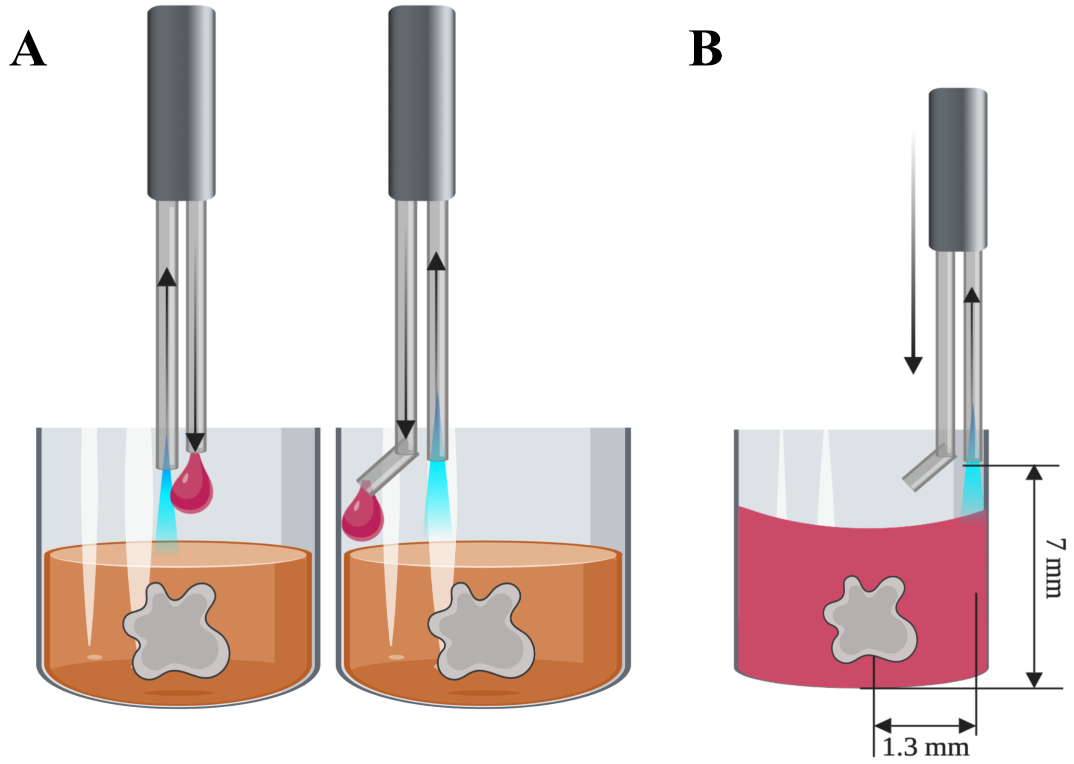

Suppl. Figure 1 Dispensing and aspiration medium with WellWash Versa. (A) Schema of dispensing the liquid with different wash heads: 1x8 wash head (on the left) and 2x8 cell wash head (on the right). In WellWash Versa robot aspiration pump is continuously working during the whole operation process, which occurred to be a very important feature in many cases. When dispensing small volumes liquid tends to form drops at the tip of the dispenser and when aspiration nozzle is located close by (on the left), it causes undesirable aspiration which causes inconsistency in volume added to the wells. This problem is solved when using 2x8 cell wash head for liquid change (on the right) where dispensing and aspiration nozzles are spatially separated. (B) Provided the fact that aspiration is performed constantly in this machine, and aspiration volume can't be controlled, height for aspiration is appearing to be a very important parameter, as It directly determines aspiration volume. Having control on the aspiration height instead of aspiration volume makes the aspiration process more consistent, because it eliminates the difference between the wells in the center of a plate and on a side where evaporation happens more effectively. Aspiration height (7 mm) was determined to match a leftover volume with already established protocol. Also, we tested different offset positions for aspiration and found out that aspirating on a side of a well (1.3 mm from the center) appeared to be more consistent than aspirating in the center due to concave meniscus shape, liquid tends to flow into the aspirator when it is going down to the well when aspirator is on a side rather than being sucked at once in the center, because the aspirator immediately destroys the surface of the liquid.
