# Supplementary material for: Semi-Automated Approach for Retinal Tissue Differentiation
Source: Transl Vis Sci Technol. 2020 Sep 23;9(10):24. doi: 10.1167/tvst.9.10.24 (PMC7521179; doi:10.1167/tvst.9.10.24)
Supplement: Supplement 2 [file tvst-9-10-24_s002.pdf]

Suppl. Table 1 Culture medium composition

	MES	OV	OC
DMEM/F12 solution (Gibco)	500ml	500ml	500ml
Sodium pyruvate 100 mM (Sigma)	5.5 ml	5.2 ml	5.2 ml
Non-essential amino acids (Gibco)	5.5 ml	5.2 ml	5.2 ml
Antibiotic-Antimycotic (Gibco)	5.5 ml	5.2 ml	5.2 ml
Chemically defined lipid concentrate (Gibco)	-	5.2 ml	5.2 ml
Fetal Bovine Serum (Gibco)	55 ml	7.6 ml	-
LIF	1000 UI/ml	-	-
Insulin Transferrin Selenium-Ethanolamine (ITS-X 100X) (Gibco)	-	1 ml	-
N-Acetyl-L-Cystein (NAC) 0.5mM stock solution	-	1.25 ml	1.25 ml
b-Mercaptoethanol 16M (Sigma)	4 ul	4 ul	4 ul
NS21 supplement	-	-	10 ml
Forskolin	-	10 uM	10 uM
Retinoic Acid (Sigma)	-	-	0.5 uM
Taurine (Sigma)	-	-	1 mM
Retinoic Acid Receptor Antagonist (Tocris)	-	1 uM	-
