# Supplementary material for: Semi-Automated Approach for Retinal Tissue Differentiation
Source: Transl Vis Sci Technol. 2020 Sep 23;9(10):24. doi: 10.1167/tvst.9.10.24 (PMC7521179; doi:10.1167/tvst.9.10.24)
Supplement: Supplement 3 [file tvst-9-10-24_s003.pdf]

Suppl. Table 2 Buffers composition

**Blocking Buffer**	
PBS	1x
Goat serum	10 %
Triton-X	0.25 %
Tween-20	0.25 %
Sodium Citrate	0.1 %
Bovine Serum Albumin	1 %
**Staining Buffer**	
PBS	1x
Triton-X	0.25 %
Tween-20	0.25 %
Bovine Serum Albumin	1 %
**Washing Buffer**	
PBS	1x
Triton-X	0.1 %
Tween-20	0.1 %
**Papain Activation Buffer**	
HBSS	50 ml
EDTA (0.5M)	110 ul (1.1mM)
L-cysteine Hydrochloride	0.043 g (5.5mM)
b-MercaptoEthanol (14.2M)	1 ul (0.3mM)
