# Supplementary material for: Semi-Automated Approach for Retinal Tissue Differentiation
Source: Transl Vis Sci Technol. 2020 Sep 23;9(10):24. doi: 10.1167/tvst.9.10.24 (PMC7521179; doi:10.1167/tvst.9.10.24)
Supplement: Supplement 4 [file tvst-9-10-24_s004.pdf]

Suppl. Table 3 Primary antibodies

Antibody	Company	Species	Cat #	Dilution
anti-Rx	Santa-Cruz	rbt	SC-79031	1/200
anti-PAX6	Hybridoma Bank	ms IgG1	AB528427	1/200
anti-Recoverin	Millipore	rbt	AB5585	1/600
Anti-Cone Arrestin	Chemicon	rbt	AB15282	1/200
anti-GS	Abcam	rbt	Ab49873	1/200
anti-PKC A	Santa-Cruz	ms IgG1	SC-8393	1/200
anti-RBPMS	Abcam	rbt	ab194213	1/200
anti-RPE65	M.Redmond	rbt		1/400
